# Psychometric properties of the Arabic Motives for Online Gaming Questionnaire

**DOI:** 10.3389/fpsyt.2026.1936722

**Published:** 2026-08-20

**Authors:** Mazen Omar Almulla, Ahmed Alduais, Abdullah Ahmed Almulla

**Affiliations:** 1 Education and Psychology, College of Education, King Faisal University, Al-Ahsa, Saudi Arabia; 2 Department of Psychology, Norwegian University of Science and Technology, Trondheim, Norway; 3 Special Education, College of Education, King Faisal University, Al-Ahsa, Saudi Arabia

**Keywords:** Arabic language, gaming disorder, gaming motive, internet gaming disorder, measurement invariance, online gaming

## Abstract

**Introduction:**

The Motives for Online Gaming Questionnaire (MOGQ) is a widely used instrument for assessing seven motivational domains underlying gaming behavior, yet no validated Arabic version exists despite the widespread use of Arabic across the Middle East and North Africa and the rapid growth of digital gaming in these regions. This study validated an Arabic version of the MOGQ among Saudi online gamers.

**Methods:**

Following International Test Commission guidelines, the MOGQ was translated into Arabic through expert panel consensus. A sample of 303 Saudi gamers (60% female; M age = 29.79 years, SD = 8.83; age range: adolescents to middle-aged adults; 60% from Eastern Region; 54% bachelor's degree; 50% single; 42% employed) completed the Arabic MOGQ and the Internet Gaming Disorder Scale–Short Form. Data were analyzed using descriptive statistics, reliability analyses, exploratory factor analysis (EFA; n = 150), confirmatory factor analysis (CFA; n = 153), concurrent validity correlations, and multi-group measurement invariance testing.

**Results:**

The Arabic MOGQ demonstrated excellent internal consistency for the total scale (α = .96, ω = .96) and satisfactory to strong reliability for all seven subscales (α range: .78–.85). An exploratory two-factor structure, explaining 49.20% of variance and corroborated by parallel analysis, emerged in the calibration subsample, distinct from a separate confirmatory test of the theoretically hypothesized seven-factor model conducted in an independent validation subsample; the seven-factor model was retained, though with modest fit indices falling below several conventional benchmarks (CFI = .84, TLI = .82, RMSEA = .10, SRMR = .08) and significant standardized factor loadings (.58–.85). All motives correlated significantly with gaming disorder symptoms (r range: .29–.62), with fantasy, social, and escape showing the strongest associations. Measurement invariance testing established metric and scalar invariance across gender; residual/strict invariance could not be separately tested under the ordinal specification.

**Discussion:**

The Arabic MOGQ is a reliable and valid instrument for assessing gaming motivation among Saudi online gamers, providing an empirical foundation for cross-cultural research and culturally informed clinical practice across Arabic-speaking populations, pending further validation in other national and regional contexts.

## 1 Introduction

Online gaming is a widespread and increasingly complex digital activity, with participation spanning adolescents and adults across diverse sociocultural contexts (1–5). Research has demonstrated that individuals engage in online gaming for multiple psychological reasons, and these motives represent a key mechanism linking gaming behavior to both adaptive outcomes and problematic engagement (6–11). The Motives for Online Gaming Questionnaire (MOGQ) has become one of the most widely used frameworks for assessing seven primary motivational domains: social interaction, escapism, competition, coping, skill development, fantasy, and recreation, and it has been validated across multiple cultural contexts and populations (2, 4, 5, 12–15).

These motives have been documented across clinical, community, and gaming samples and across different cultural contexts, highlighting the multidimensional nature of gaming engagement (1–7, 9, 10, 15). Studies consistently show that social and recreational motives are typically associated with positive engagement and enjoyment, whereas escape, coping, and sometimes competition are more strongly linked to problematic use patterns, emotional regulation difficulties, and behavioral symptoms resembling gaming disorder (8, 11, 12, 14).

Beyond the MOGQ structure, alternative theoretical models and empirical research further illustrate that online gaming motivation is dynamic and context dependent. Broader motivational frameworks, such as achievement–social–immersion models, emphasize goal attainment, relational connection, and psychological immersion as key dimensions of gaming behavior (11, 13, 16). Other work has identified motives such as exploration, aggression, professional aspiration, leisure, community, and identity-related needs, suggesting that motivational drivers are shaped by both individual characteristics and game environments (17–20). Longitudinal and qualitative research further indicates that motives may evolve over time, with shifts in life circumstances, emotional states, gaming experience, and social contexts influencing the reasons individuals continue or escalate gaming involvement (4, 12). This expanding body of literature highlights that online gaming motives are not uniform; rather, they form complex motivational profiles that differentiate casual users from high-risk or gaming disorder populations (2, 10).

Importantly, accumulating evidence demonstrates that specific motives play a critical role in predicting problematic or excessive gaming patterns, providing valuable insight into mechanisms underlying potential harm. Escapism and coping motives are consistently among the strongest predictors of excessive play, impaired control, and psychosocial difficulties, often mediating relationships between stress, emotional distress, and problematic gaming behavior (5, 8, 10, 14, 17). Competitive, achievement-oriented, and professional development motives also appear relevant, particularly for individuals engaged in esports or high-performance gaming trajectories (5, 12). Conversely, recreational and social motives are more commonly associated with positive engagement without clinically significant harm (4, 7). Together, this evidence underscores the importance of reliable and culturally valid tools such as the MOGQ to identify motivational patterns, inform prevention and intervention strategies, and advance understanding of how gaming motives contribute to psychological well-being and gaming-related risks across populations (1, 3, 9, 13, 15).

## 2 The present study

Since its development in a large Hungarian online gamer sample, the MOGQ has been translated and validated in multiple cultural and linguistic contexts, including Chinese, Iranian (Persian), Turkish, Korean, Spanish, Swedish, and Japanese versions, as well as in a game-specific sample of League of Legends players (2, 21–28). Across these studies, the MOGQ has shown acceptable factor structures, good internal consistency, and meaningful associations with gaming-related outcomes, including gaming disorder symptoms. However, no validated Arabic version is currently available, despite Arabic being the first language in roughly 22–25 states in the Middle East and North Africa and the rapid growth of digital gaming in these settings. Empirical estimates place internet gaming disorder prevalence at approximately 8.8–21.5% among Saudi university samples and considerably higher, 20–45%, among Saudi adolescent samples (29–31), underscoring the clinical and public-health relevance of a psychometrically sound Arabic gaming-motive measure. The present study addresses this gap by translating and culturally adapting the MOGQ into Arabic and examining its psychometric properties among Saudi online gamers, including item distributions and reliability, exploratory and confirmatory factor analyses of the seven-factor model, convergent validity with Internet Gaming Disorder symptoms, and measurement invariance across gender.

Based on these aims, four hypotheses were formulated: (H1) the Arabic MOGQ will exhibit a seven-factor structure in confirmatory factor analysis consistent with the original theoretical model, with standardized factor loadings exceeding.40; (H2) the Arabic MOGQ total scale and its seven subscales will demonstrate acceptable to excellent internal consistency (α ≥.70); (H3) all seven MOGQ motive subscales will show significant positive correlations with Internet Gaming Disorder symptom severity; and (H4) the Arabic MOGQ will demonstrate at least metric measurement invariance across gender.

## 3 Methods

### 3.1 Design

This study adopted a cross-sectional psychometric validation design to examine the structural validity, internal consistency, concurrent validity, and measurement invariance of the MOGQ. Psychometric validation followed recommended methodological standards for instrument evaluation, beginning with examination of descriptive item characteristics and reliability, followed by exploratory factor analysis (EFA) for structure identification and confirmatory factor analysis (CFA) for structure verification in an independent subsample (32–34). Model adequacy was evaluated using widely accepted fit indices and criteria from structural equation modeling literature (35). Concurrent validity was assessed through associations with a theoretically related construct. Finally, measurement invariance across gender was tested using hierarchical multi-group CFA to determine whether the instrument measured constructs equivalently across groups, consistent with international recommendations for fairness and comparability in psychological measurement (36, 37).

### 3.2 Sample

The sample size was determined using the participants-per-item heuristic, which commonly recommends between 5 and 10 respondents per item, with minimum benchmarks of approximately 100 cases for reliability assessment, 200 for EFA, and 300 for CFA or structural equation modeling (SEM) (38–41). Given that the Arabic MOGQ comprises 27 items, recommended thresholds were at least 100 participants for reliability, 200 for EFA, and 300 for CFA/SEM. The final sample of 303 participants met and exceeded these criteria at the full-sample level. However, because the sample was subsequently split for cross-validation purposes, the calibration subsample used for EFA (n = 150) and the validation subsample used for CFA (n = 153) are each smaller than the full sample and fall short of the 200 and 300 thresholds cited above when considered independently; this is addressed further in the Limitations section. Detailed characteristics of the sample are provided in the results section 4.1.

Participants were recruited via an online survey administered through Google Forms and distributed through professional networks, including groups of colleagues working in schools and universities across different regions of Saudi Arabia. Inclusion required current engagement in online gaming and residence in Saudi Arabia; no minimum weekly gaming-hours threshold was applied. Participants under 18 years of age (n = 17) were included only with parental or guardian consent obtained in addition to their own assent.

This study forms part of a coordinated research program using the same community-recruited Saudi sample (N = 303) to evaluate distinct, independently validated instruments. Companion analyses drawing on this dataset have examined the psychometric properties of the Arabic Internet Gaming Disorder Scale–Short Form and an unvalidated anger screener (42), and the roles of anger and social anxiety as predictors of gaming-disorder severity (43). The present study is distinct in its aims (validating the Arabic MOGQ motive structure rather than a criterion or predictor measure), its primary instrument (the 27-item MOGQ rather than the IGDS9-SF or ancillary screeners), and its analytic focus (exploratory and confirmatory factor analysis and measurement invariance of gaming motives, rather than concurrent or predictive validity of IGD or anger/social anxiety). No analyses, results, or conclusions are duplicated across these papers.

### 3.3 Measures

The Motives for Online Gaming Questionnaire (MOGQ) is a 27-item self-report instrument developed to assess seven motivational dimensions underlying online gaming behavior: Social, Escape, Competition, Coping, Skill Development, Fantasy, and Recreation (21). Items are rated on a 5-point Likert scale ranging from 1 (almost never/never) to 5 (almost always/always), with higher scores reflecting stronger endorsement of each motive. In the original large-scale validation study, based on the full sample of 3,818 respondents, the seven motivational dimensions were moderately to strongly interrelated, with factor correlations ranging from.198 to.604, all statistically significant at p <.001, indicating that the motives represent related but empirically distinct constructs (21; **Table 1**).

**Table 1:** Standardized factor loadings for the seven-factor CFA model of the Arabic MOGQ (n = 153).

Factor	Item	Loading	SE	95% CI lower	95% CI upper	Std. loading
Social	MOGQ Item 1	0.71108	0.09521	0.52447	0.89768	0.57881
	MOGQ Item 8	1.00223	0.08585	0.83397	1.17049	0.81950
	MOGQ Item 15	0.87100	0.10341	0.66831	1.07369	0.64922
	MOGQ Item 22	1.01705	0.09009	0.84049	1.19362	0.79583
Escape	MOGQ Item 2	0.80122	0.08993	0.62496	0.97748	0.65706
	MOGQ Item 9	1.04774	0.08468	0.88177	1.21370	0.83228
	MOGQ Item 16	1.07335	0.09011	0.89674	1.24996	0.80940
	MOGQ Item 23	0.98837	0.09152	0.80899	1.16774	0.75888
Competition	MOGQ Item 3	0.79324	0.09763	0.60189	0.98459	0.61947
	MOGQ Item 10	1.04810	0.10611	0.84013	1.25606	0.72128
	MOGQ Item 17	0.92254	0.09628	0.73384	1.11123	0.70671
	MOGQ Item 24	0.79024	0.09980	0.59463	0.98585	0.61170
Coping	MOGQ Item 4	0.74759	0.09241	0.56646	0.92871	0.61239
	MOGQ Item 11	0.95057	0.09466	0.76504	1.13610	0.72657
	MOGQ Item 18	0.99073	0.09479	0.80494	1.17652	0.74372
	MOGQ Item 25	1.04039	0.08794	0.86804	1.21274	0.80708
Skill development	MOGQ Item 5	0.85568	0.09082	0.67768	1.03369	0.68707
	MOGQ Item 12	0.98154	0.09603	0.79333	1.16975	0.73376
	MOGQ Item 19	1.11116	0.09683	0.92138	1.30093	0.80142
	MOGQ Item 26	0.90519	0.09959	0.71000	1.10039	0.67476
Fantasy	MOGQ Item 6	0.90063	0.08492	0.73419	1.06708	0.75307
	MOGQ Item 13	0.94188	0.08797	0.76946	1.11429	0.75411
	MOGQ Item 20	1.08045	0.08956	0.90492	1.25599	0.82015
	MOGQ Item 27	1.04573	0.09583	0.85791	1.23355	0.76282
Recreation	MOGQ Item 7	1.13933	0.09461	0.95390	1.32477	0.83137
	MOGQ Item 14	1.21026	0.09822	1.01776	1.40276	0.84638
	MOGQ Item 21	1.15292	0.10383	0.94943	1.35642	0.79106

All factor loadings are statistically significant at p <.001.

The MOGQ was translated and culturally adapted into Arabic following the International Test Commission Guidelines for Translating and Adapting Tests (Second Edition, 44). The process emphasized conceptual and functional equivalence rather than literal wording. Three bilingual experts with specializations in psychology and special education independently translated the instrument into Arabic, ensuring linguistic accuracy, clarity, and cultural appropriateness. The three translated versions were then reconciled into a unified draft through expert panel review, during which discrepancies were discussed and resolved by consensus. Particular attention was given to preserving construct meaning, ensuring the clarity of item wording and instructions, and maintaining consistency in the response scale format, in line with ITC recommendations regarding expert involvement, cultural relevance, and adaptation quality. The final Arabic item wording, instructions, response scale, and scoring procedure are available from the corresponding author upon request.

Concurrent validity was assessed using the Internet Gaming Disorder Scale–Short Form (IGDS9-SF; 45), a nine-item self-report measure of DSM-5 gaming-disorder symptoms rated on a five-point scale, with higher total scores reflecting greater symptom severity. In the present sample, the IGDS9-SF demonstrated strong reliability (α = .78, ω = .92) and a unidimensional factor structure supported by confirmatory factor analysis, with metric and scalar invariance established across sex, as reported for this same dataset in Almulla et al. (42). The Arabic IGDS9-SF has additionally undergone independent psychometric validation in a separate Saudi university-student sample, similarly supporting a unidimensional structure and adequate internal consistency (46).

### 3.4 Statistical analysis plan

Data were collected over a three-month period between August and October 2025. Ethical approval for the study protocol was obtained from the Research Ethics Committee at King Faisal University (Ref. No. KFU-REC-2025-OCT-ETHIC-S3520). Data collection began following departmental clearance to proceed, obtained during IRB submission, consistent with King Faisal University’s standard two-stage approval procedure in which formal IRB certification is issued after an initial administrative review period. All participants were informed about the purpose of the study, confidentiality assurances, voluntary participation, and the right to withdraw at any time. Written informed consent was obtained from all adult participants. For the 17 participants below the legal age, informed consent was obtained from their parents or legal guardians prior to participation. Parents/guardians were provided with a written information sheet detailing the study purpose, procedures, potential risks and benefits, confidentiality safeguards, and the voluntary nature of participation. Only after they had the opportunity to review the materials and ask questions was written informed consent obtained.

Descriptive statistics, reliability analyses, exploratory and confirmatory factor analyses, and concurrent validity correlations were conducted using jamovi 2.3 and R version 4.1, with dedicated psychometric and structural equation modeling packages (47, 48). Measurement invariance testing was subsequently re-specified and re-estimated in R version 4.3.3 using lavaan and semTools, as detailed below. First, preliminary descriptive statistics were computed for all MOGQ items and subscales, including means, standard deviations, confidence intervals, skewness, kurtosis, and Shapiro–Wilk normality tests to evaluate distributional characteristics and suitability for factor analysis. Internal consistency reliability was then examined using multiple indices, including Cronbach’s alpha and McDonald’s omega, along with item–rest correlations, using the *psych* package to ensure robust reliability estimation for multidimensional scales (49). To establish construct validity, the sample was randomly divided, with Exploratory Factor Analysis (EFA) conducted on the calibration subsample using principal axis factoring with oblique rotation to examine factorial structure and factor solutions. Confirmatory Factor Analysis (CFA) was performed on the validation subsample using DWLS estimation to evaluate the theoretically expected seven-factor model, with model adequacy assessed using χ², CFI, TLI, RMSEA, and SRMR, implemented via *lavaan* and visualized using *semPlot* (50, 51). Concurrent validity was examined through Pearson correlations between MOGQ motives and IGDS9-SF scores. Finally, measurement invariance across gender was evaluated using multi-group CFA and a hierarchical sequence of constraints (configural, metric, scalar, and strict invariance), with changes in χ² and CFI used to determine invariance adequacy. Initial models were run using SEMLj within jamovi (52); the final reported analysis was re-specified and re-estimated in R version 4.3.3 using lavaan and semTools under delta parameterization, as detailed in Section 4.7.

## 4 Results

### 4.1 Participant characteristics

Participants represented a broad range of developmental stages, educational backgrounds, and social circumstances (see **Table 2**). The estimated average age indicated that the sample predominantly comprised individuals in early to mid-adulthood. Females were more highly represented than males, and the majority resided in the Eastern Region. Most participants reported at least a bachelor’s degree, and nearly half were single, with a considerable proportion married. Employment characteristics reflected a mixed sample including working adults, students, and unemployed individuals. To minimize bias and reduce capitalization on sampling variance, the total sample was randomly divided into two independent subsamples for model development and validation procedures, while the full sample was used for reliability analyses and measurement invariance testing.

**Table 2:** Participant characteristics (N = 303).

Variable	Category	n	%
Age (estimated)	M_age_ = 29.79, SD_age_ = 8.83		
Gender	Female	182	60.07
	Male	121	39.93
Age category	Adolescents	17	5.61
	Emerging adults	82	27.06
	Young adults	69	22.77
	Adults	89	29.37
	Middle-aged adults	46	15.18
Region	Eastern Region	181	59.74
	Central Region	42	13.86
	Western Region	35	11.55
	Southern Region	23	7.59
	Northern Region	22	7.26
Educational level	High school diploma or less	92	30.36
	Bachelor’s degree	163	53.80
	Master’s degree	38	12.54
	Doctorate degree	10	3.30
Marital status	Single	150	49.51
	Married	123	40.59
	Divorced	22	7.26
	Widowed	8	2.64
Employment status	Employed	127	41.91
	Self-employed	23	7.59
	Student	89	29.37
	Unemployed	64	21.12

### 4.2 Descriptive statistics

Descriptive statistics were calculated for all 27 MOGQ items, including central tendency (mean), dispersion (standard deviation and standard error), estimation precision (95 percent confidence intervals), distributional indices (skewness and kurtosis), and normality testing using the Shapiro–Wilk test (see **Table 3**). Item means ranged from 1.99 to 3.16, indicating that responses generally clustered around low-to-moderate endorsement levels, with some items reflecting comparatively higher motivation for gaming (e.g., enjoyment-related items). Standard deviations ranged from 1.18 to 1.43, indicating substantial variability in participants’ responses, while standard errors were consistently small (0.07–0.08), reflecting stable mean estimates. The 95 percent confidence intervals around item means were relatively narrow, typically spanning approximately 0.25 to 0.32 scale points, suggesting precise estimates. Skewness values ranged from −0.16 to 0.89, indicating primarily slight positive asymmetry, whereas kurtosis values ranged from approximately −1.20 to −0.47, suggesting relatively flatter than normal distributions. Shapiro–Wilk coefficients ranged from 0.76 to 0.89, with all normality tests statistically significant (p <.001), indicating deviation from perfect normality; however, given the large sample size, these deviations are not unexpected and do not indicate problematic distributions for subsequent psychometric analyses.

**Table 3:** Descriptive statistics for the 27 MOGQ items (N = 303).

Item	M	SD	SE	95% CI Lower	95% CI Upper	Skewness	Kurtosis	W
1	1.99	1.25	0.07	1.85	2.13	0.89	−0.48	0.76
2	2.43	1.21	0.07	2.29	2.57	0.27	−0.80	0.85
3	2.49	1.27	0.07	2.35	2.64	0.30	−0.88	0.86
4	2.74	1.19	0.07	2.61	2.88	0.00	−0.67	0.88
5	2.31	1.25	0.07	2.17	2.45	0.45	−0.84	0.84
6	2.05	1.24	0.07	1.91	2.19	0.80	−0.47	0.78
7	3.16	1.35	0.08	3.00	3.31	−0.16	−1.04	0.89
8	2.14	1.27	0.07	1.99	2.28	0.63	−0.83	0.79
9	2.36	1.27	0.07	2.21	2.50	0.38	−0.89	0.83
10	2.61	1.43	0.08	2.45	2.78	0.29	−1.20	0.86
11	2.53	1.28	0.07	2.39	2.68	0.27	−0.92	0.86
12	2.54	1.31	0.08	2.39	2.69	0.26	−1.00	0.87
13	2.03	1.26	0.07	1.89	2.17	0.85	−0.47	0.77
14	3.02	1.39	0.08	2.87	3.18	−0.05	−1.13	0.88
15	2.57	1.32	0.08	2.43	2.72	0.30	−0.93	0.87
16	2.32	1.31	0.08	2.18	2.47	0.50	−0.89	0.83
17	2.24	1.32	0.08	2.09	2.39	0.56	−0.88	0.80
18	2.37	1.29	0.07	2.22	2.51	0.46	−0.83	0.84
19	2.52	1.32	0.08	2.37	2.67	0.38	−0.88	0.86
20	2.16	1.29	0.07	2.02	2.31	0.76	−0.53	0.80
21	3.08	1.40	0.08	2.92	3.23	−0.08	−1.15	0.88
22	2.25	1.31	0.08	2.10	2.40	0.58	−0.81	0.81
23	2.11	1.26	0.07	1.97	2.26	0.73	−0.58	0.79
24	2.19	1.32	0.08	2.05	2.34	0.71	−0.66	0.81
25	2.44	1.31	0.08	2.29	2.59	0.43	−0.83	0.85
26	2.34	1.31	0.08	2.19	2.48	0.52	−0.80	0.83
27	2.23	1.34	0.08	2.08	2.38	0.68	−0.75	0.81

CI, confidence interval. Shapiro–Wilk test values indicate deviation from normality across items. Shapiro–Wilk tests indicated significant non-normality for all MOGQ items, with *p* <.001 for every item.

### 4.3 Reliability

Section 4.1 established that the sample was sufficiently large, diverse, and demographically balanced to support robust psychometric testing of the Arabic MOGQ. Section 4.2 then demonstrated that the item distributions were informative, showed adequate variability, and reflected meaningful response spread across the scale, despite statistically significant deviations from perfect normality that were expected given the large sample size. Together, these findings indicate that the dataset is suitable for psychometric evaluation and justify proceeding to formal reliability assessment to examine the internal coherence of items before advancing to factor-analytic validation.

Reliability analysis showed that the full 27-item Arabic MOGQ demonstrated excellent internal consistency, with Cronbach’s alpha of.96 and McDonald’s omega of.96, indicating highly consistent responding across items. Item–rest correlations ranged from.50 to.72, confirming that all items contributed meaningfully to the construct, and alpha values if items were deleted remained within.95 to.95, indicating structural stability and no psychometric justification for item removal. Subscale reliability indices were satisfactory to strong: Social (α = .79, ω = .80), Escape (α = .83, ω = .83), Competition (α = .78, ω = .79), Coping (α = .79, ω = .79), Skill Development (α = .80, ω = .80), Fantasy (α = .83, ω = .84), and Recreation (α = .84, ω = .85). These values indicate consistent measurement precision across the seven motivational domains, supporting retention of all items as adequate contributors to their respective constructs (see **Table 4**).

**Table 4:** Internal consistency reliability of the Arabic MOGQ full scale and subscales (N = 303).

*(Sub) Scale	k	M	SD	Cronbach’s alpha	McDonald’s omega	Alpha if item deleted, range
Total MOGQ	27	2.42	0.88	0.96	0.96	0.953 to 0.955
Social	4	2.24	1.01	0.79	0.80	0.70 to 0.79
Escape	4	2.31	1.03	0.83	0.83	0.77 to 0.82
Competition	4	2.39	1.04	0.78	0.79	0.70 to 0.75
Coping	4	2.52	0.99	0.79	0.79	0.71 to 0.77
Skill development	4	2.43	1.02	0.80	0.80	0.72 to 0.76
Fantasy	4	2.12	1.05	0.83	0.84	0.76 to 0.81
Recreation	3	3.08	1.20	0.84	0.85	0.76 to 0.80

The MOGQ consists of 27 items organized into seven subscales: Social (items 1, 8, 15, 22), Escape (items 2, 9, 16, 23), Competition (items 3, 10, 17, 24), Coping (items 4, 11, 18, 25), Skill Development (items 5, 12, 19, 26), Fantasy (items 6, 13, 20, 27), and Recreation (items 7, 14, 21). Higher scores indicate stronger endorsement of the respective gaming motive.

k = number of items. All coefficients indicate acceptable to excellent internal consistency. “α if item dropped” values indicate that reliability would not meaningfully improve by removing any item.

### 4.4 Exploratory factor analysis

Based on preliminary descriptive and reliability evidence, an exploratory factor analysis was conducted to examine the latent structure of the Arabic MOGQ using the calibration subsample. To avoid capitalizing on sample-specific variance and to strengthen construct validation, the full sample (N = 303) was randomly divided into two independent subsamples. The first subsample (n = 150) was used for EFA to identify the underlying factor structure, while the second subsample (n = 153) was reserved for confirmatory testing in a subsequent CFA. This split-sample strategy is recommended in psychometric validation studies to reduce model overfitting, enhance cross-validation, and provide stronger evidence of structural validity.

**Figure 1:**
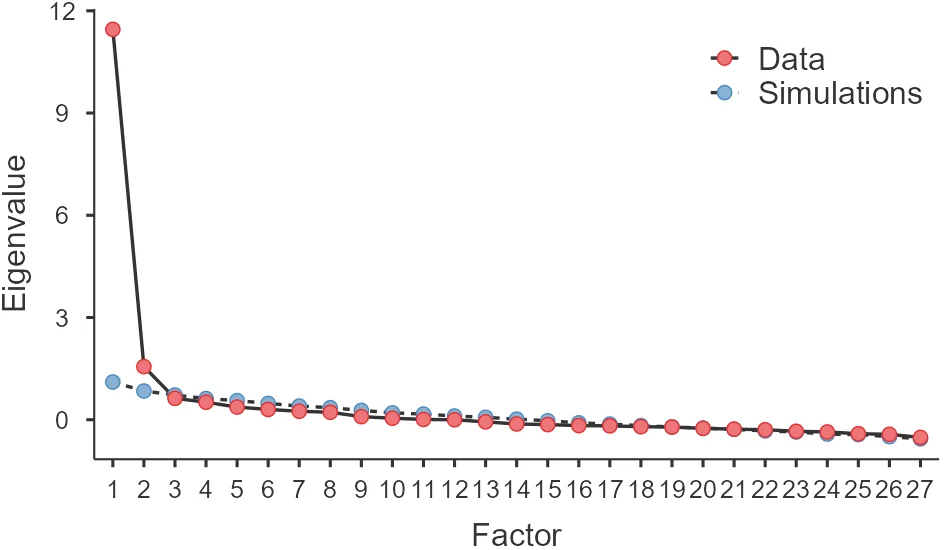
Parallel analysis scree plot for the exploratory factor analysis of the Arabic MOGQ (n = 150). Red markers show observed eigenvalues from the sample data; blue markers show mean eigenvalues from 500 simulated datasets of random data with the same sample size and number of variables. Only the first two observed eigenvalues exceed the corresponding simulated values, supporting retention of two factors.

Principal axis factoring with oblimin rotation was applied given theoretical expectations of correlated motivational constructs (**Table 5**). Sampling adequacy indices confirmed suitability for factor extraction, with an excellent Kaiser–Meyer–Olkin value of.93 and a statistically significant Bartlett’s test of sphericity, χ²(351) = 2443.50, p <.001, indicating substantial inter-item correlations. Eigenvalues greater than one and inspection of the scree plot supported a two-factor solution, a decision corroborated by Horn’s parallel analysis (500 simulated datasets, principal axis extraction): only the first two observed eigenvalues (11.46, 1.56) exceeded their corresponding simulated 95th-percentile values, with observed and simulated eigenvalues converging from the third factor onward (see **Figure 1**). Factor 1 accounted for 35.68 percent of variance and Factor 2 accounted for 13.52 percent, yielding a cumulative variance of 49.20 percent (see **Table 5**). Model adequacy indicators demonstrated acceptable fit (RMSEA = .07, 90 percent CI [.06,.08]; TLI = .87), and the factors were moderately correlated (r = .48), indicating related but distinguishable construct dimensions.

**Table 5:** Exploratory factor analysis factor loadings, uniqueness, and variance explained for the Arabic MOGQ (n = 150).

Item	Factor 1	Factor 2	Uniqueness
MOGQ 1	.63	–	.64
MOGQ 2	.60	–	.50
MOGQ 3	.40	.42	.50
MOGQ 4	.41	.40	.52
MOGQ 5	.50	–	.56
MOGQ 6	.62	–	.64
MOGQ 7	–	.81	.40
MOGQ 8	.64	–	.54
MOGQ 9	.64	–	.59
MOGQ 10	.31	.42	.60
MOGQ 11	.55	–	.58
MOGQ 12	.41	.43	.48
MOGQ 13	.86	–	.37
MOGQ 14	–	.79	.38
MOGQ 15	.49	–	.53
MOGQ 16	.75	–	.46
MOGQ 17	.65	–	.46
MOGQ 18	.67	–	.57
MOGQ 19	.49	.37	.44
MOGQ 20	.76	–	.48
MOGQ 21	–	.74	.42
MOGQ 22	.79	–	.43
MOGQ 23	.80	–	.45
MOGQ 24	.54	–	.55
MOGQ 25	.50	–	.61
MOGQ 26	.67	–	.50
MOGQ 27	.67	–	.50

Factor statistics:.

Factor 1: SS loadings = 9.63, Variance explained = 35.68%.

Factor 2: SS loadings = 3.65, Variance explained = 13.52%.

Total variance explained = 49.20%.

Loadings ≥.40 were considered salient. Extraction method: principal axis factoring. Rotation: oblimin. RMSEA = .07 (90% CI [.06,.08]); TLI = .87. Bartlett’s test: χ²(351) = 2443.50, p <.001. KMO = .93.

Factor interpretation was guided by a ≥.40 loading threshold. Most items showed strong primary loadings on Factor 1 (range.41 to.86), particularly items relating to fantasy, coping, escape, skill development, and social–motivational content. A smaller cluster of items loaded clearly on Factor 2 (loadings.74 to.81), primarily reflecting recreational and enjoyment-based motives. Only a small number of items demonstrated modest cross-loadings, and no item met criteria for removal based on loading strength or uniqueness. Taken together, the EFA findings support a coherent and interpretable two-factor motivational structure for the Arabic MOGQ, providing an empirically defensible foundation for subsequent confirmatory factor analysis.

### 4.5 Confirmatory factor analysis

To cross-validate the structure suggested by the EFA and by previous MOGQ research, we conducted a CFA on the second random subsample (n = 153), specifying the original seven correlated first-order factors (Social, Escape, Competition, Coping, Skill Development, Fantasy, Recreation). This step followed the preliminary distributional checks (Section 4.2) and reliability analyses (Section 4.3), which showed that the items were reasonably distributed and highly internally consistent, and the EFA (Section 4.4), which indicated a coherent multidimensional structure. The CFA tested whether this seven-factor configuration would reproduce the observed covariance structure in an independent subsample, providing a stricter test of structural validity while preserving separate motivational domains instead of a single global score.

Model fit indices indicated a modest reproduction of the item covariances, falling below several conventional benchmarks (35), with χ²(303) = 750.88, p <.001, RMSEA = .10 (90% CI [.09,.11]), SRMR = .08, CFI = .84, and TLI = .82. All unstandardized factor loadings were statistically significant (all ps <.001), and standardized loadings were uniformly moderate to high across items, ranging from.58 to.85 (see **Table 1**). Factor correlations were substantial, with standardized covariances ranging from.49 (Fantasy–Recreation) to.98 (Escape–Coping); the complete latent factor correlation matrix is presented in **Table 6**. The Escape–Coping correlation approaching unity indicates that these two factors are not empirically distinguishable at the structural level in the present sample, and several additional pairs (Coping–Skill Development = .95, Competition–Skill Development = .94, Social–Competition = .94) also exceeded conventional discriminant-validity thresholds, indicating that discriminant validity concerns extend beyond a single factor pair (see Discussion and Limitations). Given these results, the correlated seven-factor model was retained on theoretical grounds, for consistency with the original Hungarian MOGQ framework and the majority of prior cross-cultural adaptations, and to support the study’s applied focus on subscale scores rather than a total score. However, its structural validity was not fully supported in the present sample: no alternative confirmatory models (e.g., a two-factor structure paralleling the EFA solution, or higher-order/bifactor specifications) were formally tested against the seven-factor model, and the seven-factor model should therefore not be considered empirically confirmed as the best-fitting structure for this sample.

**Table 6:** Standardized latent factor correlation matrix for the seven-factor CFA of the Arabic MOGQ (n = 153).

Factor	Social	Escape	Comp.	Coping	Skill dev.	Fantasy	Recr.
Social	—	.81	.94	.79	.81	.91	.55
Escape		—	.91	.98	.84	.91	.65
Competition			—	.89	.94	.85	.76
Coping				—	.95	.86	.68
Skill Development					—	.88	.78
Fantasy						—	.49
Recreation							—

Values are standardized factor covariances (i.e., latent correlations) from the confirmatory factor analysis; all correlations were statistically significant at p <.001. The highest correlation (Escape–Coping = .98) and several other pairs at or above.90 (Social–Competition = .94, Competition–Skill Development = .94, Coping–Skill Development = .95, Social–Fantasy = .91, Escape–Competition = .91, Escape–Fantasy = .91) indicate limited discriminant validity among several motivational subscales.

### 4.6 Concurrent validity

Establishing concurrent validity is essential to demonstrate that the MOGQ relates to external constructs in theoretically predictable ways when measured at the same time. To evaluate this, we correlated the seven MOGQ subscales with the total score of the Internet Gaming Disorder Scale–Short Form (IGDS9-SF). All correlations were positive and statistically significant (ps <.001). The strongest associations with IGD symptoms emerged for Fantasy (r = .62, 95% CI [.54,.68]), Social (r = .57, 95% CI [.49,.65]), and Escape (r = .56, 95% CI [.48,.63]) motives. Moderate correlations were observed for Coping (r = .53, 95% CI [.44,.60]), Competition (r = .52, 95% CI [.43,.59]), and Skill Development (r = .51, 95% CI [.42,.59]). Recreation showed the weakest association (r = .29, 95% CI [.18,.39]). Overall, the pattern of correlations aligns with theoretical expectations—motives related to immersion, fantasy, and escape showed the strongest links to IGD—indicating that the MOGQ demonstrates satisfactory concurrent validity.

### 4.7 Measurement invariance across gender

Building on the findings reported in Sections 4.1 to 4.6, which established item-level distributions, internal consistency, factorial validity, and the stability of the seven-factor Arabic MOGQ structure, the next essential step was to examine whether this measurement structure functions equivalently across male and female participants. This step is necessary because meaningful comparison of latent factor scores or mean differences requires evidence that the construct is measured in the same way across groups. Without establishing measurement invariance, any observed gender differences could reflect measurement bias rather than true psychological differences (see **Table 7**).

**Table 7:** Measurement invariance for the Arabic MOGQ (revised following R/semTools reanalysis; see Statistical Analysis Plan).

Invariance	χ²	df	CFI	TLI	RMSEA	90% CI RMSEA	SRMR	Δχ²	Δdf	p (Δχ²)	ΔCFI
Configural	1106.44	606	.952	.944	.074	[.067,.081]	.070	—	—	—	—
Metric	1108.91	626	.954	.948	.072	[.065,.078]	.077	51.87	20	<.001	+.002
Scalar	1190.04	707	.954	.954	.067	[.061,.074]	.077	97.80	81	.099	.000
Strict	1190.04	707	.954	.954	.067	[.061,.074]	.077	—^a^	0	—^a^	.000

• All models estimated in R using semTools’ measEq.syntax() (delta parameterization) with lavaan/DWLS;.

χ², CFI, TLI, RMSEA, and SRMR are scaled (robust) statistics.

• Δχ², Δdf, and p for the Metric and Scalar rows are scaled chi-square difference tests relative to the preceding model.

^a^ The Strict model added no further identifiable degrees of freedom beyond the Scalar model (Δdf = 0) because item residual variances are not freely estimated under the delta parameterization used here, and is therefore not separately testable in this specification; its fit indices are identical to the Scalar model.

Measurement invariance was tested in a hierarchical framework using multi-group confirmatory factor analysis with Diagonally Weighted Least Squares (DWLS) estimation. Initial models were estimated in jamovi (SEMLj module); following reviewer feedback identifying inconsistencies in the resulting constraint sequence, invariance testing was re-specified and re-estimated in R (version 4.3.3) using the lavaan (50) and semTools (53) packages, with configural, metric, scalar, and strict models generated via semTools’ measEq.syntax() function under delta parameterization (the lavaan/semTools default for ordinal indicators), following the identification conventions for ordinal factorial invariance described by Millsap and Yun-Tein (54). This estimator and parameterization are appropriate given the ordinal nature of the 5-point item response scale and provide identification conditions suitable for testing invariance of thresholds and residual variances with categorical indicators. The seven latent factors (Social, Escape, Competition, Coping, Skill Development, Fantasy, Recreation) were specified as reflective constructs, with each factor indicated by its corresponding items (four items for each factor except Recreation, which comprised three items), consistent with the validated EFA and CFA solutions reported earlier. Configural invariance tested whether the same factor–item structure holds across groups with no equality constraints, followed by metric invariance where factor loadings were constrained equal, scalar invariance where loadings and item thresholds were additionally constrained (with latent factor means freed in the non-reference group for identification), and strict invariance where item residual (unique) variances were additionally constrained. This structured sequence allowed determination of the level at which equivalence is supported and the extent to which scale scores may be legitimately compared between males and females.

Configural invariance was first tested using a multi-group CFA across gender, specified via semTools’ measEq.syntax() with delta parameterization and DWLS estimation. The seven-factor model with freely estimated loadings and thresholds in both groups showed acceptable fit, scaled χ²(606) = 1106.44, CFI = .952, TLI = .944, RMSEA = .074, 90% CI [.067,.081], SRMR = .070, providing a suitable baseline for evaluating stronger equality constraints.

Metric invariance was examined by constraining all factor loadings to equality across gender while allowing thresholds, residuals, latent variances, and latent covariances to remain freely estimated. The metric model showed fit comparable to the configural model, scaled χ²(626) = 1108.91, CFI = .954, TLI = .948, RMSEA = .072, 90% CI [.065,.078], and SRMR = .077. The scaled chi-square difference test was statistically significant, Δχ²(20) = 51.87, p <.001; however, the change in practical fit indices was negligible and in the direction of improved fit (ΔCFI = +.002, ΔRMSEA = −.003). Because chi-square difference tests are known to be oversensitive to sample size (55), and because the widely used ΔCFI ≤.01/ΔRMSEA ≤.015 criteria were met, metric invariance was supported, indicating that males and females weighted the underlying motivational items comparably.

Scalar invariance was then tested by additionally constraining item thresholds to equality across gender, while residual variances, latent variances, latent covariances, and latent factor means (in the non-reference group) were freely estimated. The scalar model showed good fit, scaled χ²(707) = 1190.04, CFI = .954, TLI = .954, RMSEA = .067, 90% CI [.061,.074], and SRMR = .077. The scaled chi-square difference relative to the metric model was non-significant, Δχ²(81) = 97.80, p = .099, and changes in CFI and RMSEA were negligible (ΔCFI = .000, ΔRMSEA = −.005). Scalar invariance was therefore supported, indicating that item thresholds were equivalent across gender and that comparisons of latent factor means across gender are appropriate (56).

Strict invariance was evaluated by additionally constraining item residual (unique) variances to equality across gender. Under the delta parameterization used for the reported models, item residual variances for ordinal indicators are not freely estimated parameters but are instead fixed as a function of the factor loadings and total item variance; consequently, this constraint did not add any further identifiable degrees of freedom beyond the scalar model (Δdf = 0), and residual invariance could not be separately evaluated from scalar invariance within the present specification. This is a recognized limitation of delta parameterization for ordinal factorial invariance testing: theta parameterization is typically required to test residual invariance directly (54). We attempted the analysis under theta parameterization, but it did not produce a properly converged, nested solution at the scalar step with the present data (non-monotonic degrees of freedom and a negative scaled chi-square difference), and delta parameterization was therefore retained as the specification that produced a stable, interpretable, and properly nested solution through the scalar level. Consistent with this constraint, strict invariance testing is rarely reported for ordinal measures in the applied literature (56), and it is reported here as untestable in the current specification rather than as supported or rejected; substantive conclusions are accordingly limited to the metric and scalar levels.

## 5 Discussion

The present study addressed a critical gap in the cross-cultural assessment of gaming motivation by developing and validating an Arabic version of the MOGQ among Saudi online gamers. This work responds to the absence of validated Arabic-language instruments for measuring gaming motives, despite Arabic being spoken across 22–25 states in the Middle East and North Africa and the rapid expansion of digital gaming in these regions. The psychometric evaluation yielded seven key findings that collectively demonstrate the instrument’s validity and reliability. First, the sample of 303 participants exhibited adequate demographic diversity and variability in gaming patterns, providing a robust foundation for psychometric testing. Second, item-level descriptive statistics revealed appropriate response distributions with meaningful variance, indicating that items captured substantive individual differences in gaming motives. Third, internal consistency analyses demonstrated excellent reliability for both the full scale and all seven subscales, confirming that items consistently measured their intended constructs. Fourth, exploratory factor analysis identified a coherent two-factor structure accounting for nearly half the variance, suggesting underlying motivational dimensions while preserving interpretability. Fifth, confirmatory factor analysis tested the theoretically expected seven-factor model in an independent subsample; the model was retained on theoretical grounds, though its fit indices fell below several conventional benchmarks, providing only partial and qualified support for the original MOGQ structure in the Arabic context. Sixth, concurrent validity analyses revealed theoretically consistent associations between gaming motives and Internet Gaming Disorder symptoms, with fantasy, social, and escape motives showing the strongest relationships. Seventh, measurement invariance testing established metric and scalar invariance across gender (residual/strict invariance was not separately testable), indicating that the Arabic MOGQ measures gaming motives equivalently for males and females, thereby supporting meaningful cross-group comparisons and advancing the instrument’s utility for research and clinical application in Arabic-speaking populations.

The factorial structure observed in the current study aligns with and diverges from previous MOGQ validations in theoretically informative ways. The original Hungarian validation by Demetrovics et al. (21) established the seven-factor, 27-item structure through rigorous multi-stage factor analyses across large subsamples, reporting strong internal consistency and acceptable confirmatory fit indices. Our Arabic validation similarly retained all 27 items and the seven-factor configuration on theoretical grounds, with internal consistency coefficients comparable to or exceeding those in the Hungarian sample, and CFA fit indices that fell below several conventional benchmarks, indicating only modest structural reproduction of the theoretically hypothesized model. The Korean validation by Kim and Kang (26) likewise confirmed the original seven-factor solution with excellent fit and no item modifications, paralleling our retention of the full item set and theoretical structure. Similarly, the Persian validation by Hamzehzadeh et al. (23) supported the seven-factor model after minor modifications involving correlated residuals rather than item deletion, a strategy that preserved conceptual fidelity while improving empirical fit—an approach conceptually consistent with our decision to retain the theoretically grounded structure. In contrast, the Turkish study by Evren et al. (22) merged the Coping and Escape factors into a single dimension and removed one item, reflecting a six-factor solution. The Spanish validation by Infanti et al. (24) also favored a six-factor structure with Escape and Coping combined and three items removed, further supporting a bifactor model emphasizing a general gaming motivation factor. The Swedish study by Bäcklund et al. (2) removed one Recreation item due to poor loading, resulting in a 26-item, seven-factor model and additionally derived a 14-item short form. These structural variations suggest that while the core MOGQ constructs demonstrate cross-cultural robustness, certain motivational domains—particularly the distinction between escape-oriented and coping-oriented gaming—may exhibit culture-specific nuances or item-level differences in interpretation. Our retention of the original seven-factor structure positions the Arabic MOGQ as consistent with the majority of validation studies while acknowledging that future research may benefit from examining whether certain item refinements enhance measurement precision in Middle Eastern contexts.

The convergence of Escape, Coping, Fantasy, Social, and Skill Development motives onto a single broad EFA factor, while Recreation remained empirically distinct, may reflect features of the sociocultural context in which gaming occurs for Saudi adult gamers. In collectivist Gulf societies, gaming that serves affect-regulation functions—escaping negative mood states, coping with stress, seeking social connection, or engaging fantasy and mastery needs—may be experienced by users as functionally interchangeable expressions of a single underlying motivation to withdraw from or compensate for offline stressors, rather than as psychologically distinct drives, particularly where alternative leisure and mental-health outlets are comparatively limited. Recreation, by contrast, describes gaming pursued for its own enjoyment without a compensatory or affect-regulatory function, which may explain why it separated cleanly from the broader cluster. This pattern of convergence among affect-related motives has also been noted, to varying degrees, in other MOGQ validations outside the original Hungarian context (2, 12), suggesting the coalescence observed here may not be uniquely Saudi but could be more pronounced where cultural norms constrain open emotional expression and where digital leisure has rapidly outpaced the development of alternative recreational infrastructure. This interpretation remains a plausible mechanism rather than a directly tested hypothesis and should be evaluated in future qualitative or mixed-methods research with Saudi gamers.

This interpretation is directly supported by the CFA results reported above: Escape and Coping showed the highest latent factor correlation in the present sample (r = .98; **Table 6**), consistent with the EFA pattern and with prior MOGQ adaptations (e.g., Turkish, Spanish validations) that have combined these two motives into a single dimension. Several other factor pairs also correlated at or above.90, suggesting that the broader pattern of limited discriminant validity observed here is not confined to a single pair and may reflect a more general tendency for affect-regulation-related gaming motives to be less differentiated in this cultural context than in the original Hungarian framework.

The internal consistency and reliability estimates obtained in the Arabic validation are highly consistent with those reported across international samples, supporting the psychometric robustness of the instrument. Our total scale reliability coefficients (α = .96, ω = .96) closely matched the Chinese validation by Wu et al. (28), which reported a total alpha of.95, and aligned with the Hungarian study’s subscale alphas ranging from.79 to.90 (21). Subscale reliabilities in the current study ranged from.78 to.85, falling within or slightly above the ranges observed in most prior validations. For instance, the Korean validation reported subscale alphas between.83 and.91 (26), the Persian study documented alphas from.78 to.91 with strong test-retest reliability (23), and the Swedish study reported McDonald’s omega values from.77 to.93 for the 27-item version (2). Even in contexts where factor structures were modified, such as the Turkish six-factor model, reliability coefficients remained strong (.87–.92; 22), and the Spanish bifactor solution similarly yielded high subscale reliability despite item deletions (24). Notably, the League of Legends-specific validation by Memeti et al. (27) reported lower reliability for certain subscales, particularly Recreation (α = .55) and Social (α = .72), suggesting that game-specific contexts may introduce unique measurement challenges not observed in general gaming populations. The consistently high reliability of the Arabic MOGQ across all seven subscales indicates that the translated items function cohesively within each motivational domain and that respondents interpret the constructs with clarity and consistency, reinforcing confidence in the instrument’s measurement precision for Arabic-speaking gamers.

Concurrent validity findings from the Arabic MOGQ closely mirror patterns documented in previous validations, further supporting the instrument’s construct validity and theoretical relevance. In the present study, fantasy, social, and escape motives exhibited the strongest correlations with Internet Gaming Disorder symptoms, followed by moderate associations for coping, competition, and skill development, while recreation showed the weakest relationship. This pattern aligns closely with the Chinese validation by Wu et al. (28), which reported that escape and coping motives were most strongly associated with IGD symptoms and need frustration, and with the Hungarian findings where escape-related motives consistently predicted problematic gaming (21). The Turkish study similarly found that the merged Coping/Escape factor demonstrated the strongest associations with gaming disorder indicators (22), and the Spanish validation identified the Escape/Coping dimension and Fantasy as the motives most robustly linked to IGD symptoms, with Recreation largely unrelated to problematic outcomes (24). The Persian validation confirmed significant positive correlations between all MOGQ subscales and IGD, with particularly strong relationships for escape and coping motives (23). Even in the game-specific context examined by Memeti et al. (27), escape and coping motives predicted higher engagement intensity and potential risk indicators among League of Legends players. The Korean study extended these findings by demonstrating that specific motives incrementally predicted problematic gaming beyond gaming time alone (26), and the Swedish study validated the MOGQ’s utility within both WHO and APA gaming disorder frameworks, with escape and coping motives again emerging as key risk indicators (2). Collectively, these cross-cultural replications underscore a robust empirical generalization: motives reflecting psychological escape, emotional coping, and immersive fantasy consistently predict heightened risk for problematic gaming, whereas recreational motives reflect more benign engagement. The Arabic MOGQ’s replication of this pattern strengthens confidence that gaming motivation operates through similar psychological mechanisms across diverse cultural contexts, supporting the instrument’s global applicability and theoretical coherence.

Measurement invariance testing in the Arabic validation yielded findings that both converge with and extend prior research, contributing important evidence regarding the generalizability of MOGQ scores across demographic groups. The present study established metric and scalar invariance across gender, indicating that factor loadings and item thresholds functioned equivalently for males and females, thereby supporting valid comparisons of latent factor means; residual (strict) invariance could not be separately evaluated under the ordinal identification approach required here. This finding aligns with the Spanish validation by Infanti et al. (24), which documented full measurement invariance across gender for their modified six-factor bifactor solution, and with the Swedish study by Bäcklund et al. (2), which similarly reported measurement invariance across gender for both the 26-item and 14-item versions. These consistent results across Arabic, Spanish, and Swedish samples suggest that the MOGQ constructs are understood and endorsed comparably by male and female gamers, at least at the level of factor structure and item functioning, despite well-documented gender differences in gaming preferences, genre choices, and participation rates. In contrast, several earlier validations, including the original Hungarian study (21), the Chinese validation (28), the Turkish study (22), the Korean validation (26), and the Persian study (23), did not formally test measurement invariance across gender or other demographic variables, leaving questions about factorial equivalence unaddressed in those contexts. The League of Legends study by Memeti et al. (27) similarly focused on structural validation without invariance testing. The accumulating evidence from the Arabic, Spanish, and Swedish studies now provides a stronger empirical foundation for asserting that the MOGQ operates as a fair and comparable measure across gender, supporting its use in research examining gender differences in gaming motives and reducing concern that observed mean differences reflect measurement artifacts rather than true psychological variation. Future research should continue testing invariance across additional demographic and cultural groupings to further establish the MOGQ’s cross-cultural measurement equivalence and ensure that motivational constructs generalize appropriately across diverse gamer populations worldwide.

### 5.1 Limitations

Several methodological considerations warrant acknowledgment when interpreting the findings of this validation study. First, the cross-sectional design precludes conclusions about the temporal stability of gaming motives or their causal relationships with gaming outcomes, and the absence of test-retest reliability data limits our understanding of measurement stability over time. Second, although the sample size met recommended psychometric benchmarks and exhibited reasonable demographic diversity, participants were recruited primarily from the Eastern Region of Saudi Arabia and represented convenience rather than probability sampling, potentially limiting generalizability to gamers from other Arabic-speaking countries or regions with different sociocultural contexts, gaming infrastructures, or regulatory environments. Third, while the sample included both adolescents and adults across a broad age range, the small proportion of adolescent participants (5.6%) and the predominance of emerging and young adults may not adequately represent motivational patterns among younger gamers, who constitute a substantial and developmentally distinct segment of the gaming population. Fourth, the reliance exclusively on self-report measures introduces potential biases related to social desirability, recall accuracy, and shared method variance, and the concurrent validity evidence was limited to associations with a single criterion measure (IGDS9-SF) rather than a broader nomological network of constructs such as personality traits, mental health indicators, or behavioral gaming metrics. Fifth, confirmatory fit indices fell below several conventional benchmarks (CFI = .84, TLI = .82, RMSEA = .10), and the latent factor correlation matrix (**Table 6**) revealed a correlation of.98 between Escape and Coping, indicating these two factors are not empirically distinguishable in the present sample, with several additional pairs also exceeding.90; minor structural refinements, correlated error terms, or alternative modeling approaches such as bifactor or hierarchical structures might improve empirical fit and subscale separation while preserving theoretical interpretability. Sixth, following reanalysis, metric and scalar invariance across gender were both supported (see Statistical Analysis Plan and Results), indicating that factor loadings and item thresholds functioned equivalently across gender and that latent mean comparisons are appropriate; however, residual (strict) invariance could not be separately evaluated because item residual variances are not freely estimated under the delta parameterization used for the reported models, and this level of invariance is accordingly reported as untestable rather than supported. Seventh, although the full sample (N = 303) exceeded common benchmarks for reliability, EFA, and CFA/SEM, the calibration (n = 150) and validation (n = 153) subsamples used for the split-sample EFA/CFA design are each smaller than the full sample and fall below the 200 and 300 thresholds typically recommended for these analyses individually; results from these subsamples, particularly the CFA of a seven-factor, 27-indicator ordinal model, should accordingly be interpreted with some caution regarding parameter stability. Finally, the study did not examine measurement invariance across other potentially meaningful demographic variables such as age groups, educational levels, employment status, or gaming intensity categories, leaving open questions about whether the Arabic MOGQ functions equivalently across these subpopulations and whether motivational constructs exhibit differential item functioning or structural differences in more granular analyses.

### 5.2 Implications

The successful validation of the Arabic MOGQ carries important theoretical, methodological, and applied implications for gaming research and practice in Arabic-speaking regions. From a theoretical perspective, the theoretical retention of the seven-factor motivational structure (despite its incompletely supported structural validity in the present sample) and the consistent pattern of associations between specific motives and gaming disorder symptoms together contribute to the growing cross-cultural evidence base on psychological mechanisms underlying gaming behavior, while also highlighting areas where cultural context may shape the salience or interpretation of specific motivational dimensions. Methodologically, the availability of a psychometrically sound Arabic instrument enables researchers in Middle Eastern and North African contexts to conduct rigorous investigations of gaming motivation using a standardized measure comparable to those employed internationally, thereby facilitating cross-cultural comparisons, meta-analytic syntheses, and multinational collaborative research that can advance understanding of gaming behavior as a global phenomenon. The establishment of measurement invariance across gender further strengthens the instrument’s utility for examining gender differences in gaming motives with confidence that observed disparities reflect genuine psychological variation rather than measurement artifacts, supporting more nuanced investigations of how motivational profiles differ between male and female gamers and how these differences relate to participation patterns, game preferences, and risk trajectories. From an applied perspective, clinicians, educators, and public health professionals working with Arabic-speaking populations now have access to a validated tool for assessing individual differences in gaming motivation, which can inform personalized prevention strategies, tailored interventions addressing the psychological needs associated with specific motivational profiles (e.g., escape- or coping-oriented gaming). The MOGQ measures gaming motives rather than gaming disorder status, and this study did not evaluate diagnostic accuracy, clinical cutoffs, sensitivity, specificity, or predictive validity; accordingly, the instrument should not be used as a standalone screening or diagnostic tool, and the present findings are best regarded as preliminary psychometric evidence from a Saudi community sample rather than a basis for early identification of at-risk individuals or clinical risk profiling. With this caveat, the Arabic MOGQ may still provide a foundation for designing psychoeducational programs that promote awareness of motivational patterns and for evaluating interventions aimed at shifting motivational orientations from maladaptive to adaptive patterns, pending future research establishing its clinical utility. More broadly, the instrument’s validation supports the integration of Middle Eastern and North African samples into international research networks, helping to diversify the evidence base and ensure that theoretical models, diagnostic frameworks, and intervention approaches are informed by data from underrepresented populations rather than relying predominantly on Western or East Asian samples.

## 6 Conclusion

This study developed and evaluated an Arabic version of the MOGQ, providing the first psychometrically evaluated instrument for assessing gaming motivation in Arabic-speaking populations. The Arabic MOGQ demonstrated excellent internal consistency, meaningful concurrent validity with gaming disorder symptoms, and metric and scalar measurement equivalence across gender. Its factor structure was theoretically retained as seven correlated motives, consistent with the original framework and most prior adaptations, but this structure showed only modest confirmatory fit and limited discriminant validity between at least one pair of subscales in the present sample; structural validity of the seven-factor model therefore requires further confirmation, ideally through comparison with alternative factor structures in future work. The findings contribute to the expanding international literature on gaming motivation by replicating core motivational constructs and their associations with problematic gaming across a novel cultural and linguistic context, while also revealing nuanced structural considerations that may reflect cultural or methodological variations in how gaming motives are conceptualized and measured. The validation of the Arabic MOGQ addresses a critical gap in the assessment infrastructure for gaming research in the Middle East and North Africa, enabling future investigations to examine how motivational profiles relate to gaming behaviors, psychological outcomes, and disorder risk in these populations, and supporting the development of culturally informed prevention and intervention strategies tailored to the specific needs and contexts of Arabic-speaking gamers. As digital gaming continues to grow rapidly across the region, the Arabic MOGQ provides researchers, clinicians, and policymakers with an essential tool for understanding the psychological drivers of gaming engagement and for identifying individuals whose motivational patterns may place them at heightened risk for negative outcomes, ultimately advancing both scientific knowledge and public health efforts in this increasingly important domain of human behavior.

## Data Availability

The raw data supporting the conclusions of this article will be made available by the authors, without undue reservation.
